# Persistent mortality in critical COVID-19 ICU cases from wild-type to delta variant: A descriptive cohort study

**DOI:** 10.1038/s41598-025-94483-3

**Published:** 2025-03-25

**Authors:** Lars Palmowski, André Hagedorn, Andrea Witowski, Helge Haberl, Felix Kraft, Ute Achtzehn, Detlef Kindgen-Milles, Kai Zacharowski, Axel Nierhaus, Maximilian Dietrich, Valbona Mirakaj, Thea Koch, Patrick Meybohm, Michael Adamzik, Lars Bergmann, Tim Rahmel

**Affiliations:** 1 Klinik Für Anästhesiologie, Intensivmedizin und Schmerztherapie, Universitätsklinikum Knappschaftskrankenhaus Bochum, 44892 Bochum, Germany; 2 Klinische Abteilung Für Allgemeine Anästhesie Und Intensivmedizin, Medizinische Universität Wien, Währinger Gürtel 18–20, 1090 Vienna, Austria; 3 Klinik Für Innere Medizin IV, Klinikum Chemnitz gGmbH, Flemmingstraße 2, 09116 Chemnitz, Germany; 4 Klinik Für Anästhesiologie Und Intensivmedizin, Universitätsklinikum Düsseldorf, Moorenstr. 5, 40225 Düsseldorf, Germany; 5 Klinik Für Anästhesiologie, Intensivmedizin und Schmerztherapie, Universitätsklinikum Frankfurt, Goethe Universität, Theodor-Stern-Kai 7, 60590 Frankfurt am Main, Germany; 6 Klinik Für Intensivmedizin, Universitätsklinikum Hamburg-Eppendorf, Martinistraße 52, 20246 Hamburg, Germany; 7 Klinik Für Anästhesiologie, Universitätsklinikum Heidelberg, Im Neuenheimer Feld 420, 69120 Heidelberg, Germany; 8 Klinik Für Anästhesiologie Und Intensivmedizin, Universitätsklinikum Tübingen, Hoppe-Seyler-Str. 3, 72076 Tübingen, Germany; 9 Klinik Und Poliklinik Für Anästhesiologie und Intensivtherapie, Universitätsklinikum Carl Gustav Carus Dresden, Technische Universität Dresden, Fetscherstraße 74, 01307 Dresden, Germany; 10 Universitätsklinikum Würzburg, Klinik und Poliklinik für Anästhesiologie, Intensivmedizin, Notfallmedizin und Schmerztherapie, Oberdürrbacher Str. 6, 97080 Würzburg, Germany

**Keywords:** COVID-19, Pandemic, SARS-CoV-2, Critical illness, ICU mortality, Virus variants, Infectious diseases, Viral infection

## Abstract

The SARS-CoV-2 pandemic led to significant advancements in treatment and vaccination, contributing to a decline in overall COVID-19-related mortality. However, it remains unclear whether the mortality rate for critical cases treated on intensive care units has also decreased. This multicentric, multinational retrospective observational study analyzed 447 critically ill COVID-19 patients treated on ICUs across ten study centers in Germany and Austria. Patients were categorized into two periods: period 1 (March 2020 to May 2021, n = 316) and period 2 (June 2021 to January 2022, n = 131). Despite evolving treatment strategies and widespread vaccine availability in period 2, 30-day mortality remained unchanged (30% in period 1 vs. 37% in period 2; HR 1.26, 95% CI: 0.90–1.79, p = 0.181). Further outcomes, including ICU-free days (p = 0.735), ventilatory support-free days (p = 0.699), vasopressor-free days (p = 0.379), and dialysis-free days (p = 0.396), also showed no significant differences. Notably, 81% (106 of 131) of ICU patients in period 2 were unvaccinated, underscoring the persistent vulnerability of this group. These findings suggest that while public health measures reduced overall COVID-19 severity, critical illness remained highly lethal. Further research is needed to explore targeted interventions for high-risk ICU patients and to better understand the factors contributing to persistent mortality despite medical advancements.

## Introduction

The COVID-19 pandemic has resulted in almost 6 million documented deaths worldwide and has been associated with significant restrictions in daily life^1,2^. A characteristic of the pandemic has been its occurrence in several waves^3^. In the initial phase of the pandemic– during the first and second waves up to June 2021 – numerous critical cases led to shortages in intensive care unit (ICU) capacity^4,5^. These critical cases were often characterized by acute respiratory distress syndrome (ARDS) and viral sepsis, both of which were associated with concerningly high mortality rates^6–8^. As the pandemic progressed, virus mutations and the global vaccination campaign contributed to a reduced incidence of these critical cases, resulting in a notable decline in COVID-19-associated mortality beginning with the third wave in June 2021^9,10^. This trend was also reflected in intensive care, with a decrease in the number of critically ill COVID-19 patients^11^.

However, for those who did develop critical illness, the burden of disease remained exceptionally high^12^. While previous research has explored overall COVID-19 mortality, there remains limited data specifically analyzing mortality trends among ICU patients—an especially vulnerable subgroup with persistently high fatality rates despite medical advancements. Thus, it remains unclear to which extent later virus variants, the increasing immunity within the population, and overall improvements in treatment itself have contributed to a reduction in mortality in these critically ill patients^6,13^.

In this study, we aimed to address this knowledge gap by investigating whether 30-day mortality in critically ill COVID-19 patients decreased over the course of the pandemic, independent of changes in case severity. By focusing specifically on ICU patients and assessing the interplay between evolving virus variants, population immunity, and advancements in critical care, we sought to provide novel insights into the trajectory of mortality among the most severely affected individuals suffering from critical COVID-19.

## Materials and methods

### Study design and conceptual overview

This study was a multicentric, multinational retrospective observational analysis of 447 critically ill patients with Polymerase Chain Reaction confirmed SARS-CoV-2 infection, admitted to ICU. The study encompassed ten study centers across Germany and Austria (i.e. University hospitals of Bochum, Chemnitz, Dresden, Düsseldorf, Frankfurt am Main, Hamburg, Heidelberg, Tübingen, Vienna, Würzburg), and the period of data collection ranged from March 2020 to January 2022. Inclusion criteria included patients aged 18 years or older with a critical course of COVID-19, as defined by the World Health Organization (WHO) stage ≥ 5^14^. Criteria for critical illness included respiratory distress, respiratory rates of ≥ 30 breaths per minute, oxygen saturation ≤ 93% at rest in ambient air or requiring supplemental oxygen, an oxygenation index (arterial oxygen partial pressure [paO_2_]/fractional inspired oxygen [FiO_2_]) ≤ 300 mmHg or the presence of septic shock, or multiple organ dysfunction. For analysis, the study period was divided into two phases, with June 2021 chosen as the cutoff point. This marked the time when over 50% of the German and Austrian population had been fully vaccinated against SARS-CoV-2 and when the distribution of virus variants had shifted^15^. This division allows for an assessment of potential mortality differences before and after widespread vaccine coverage.

### Ethical approval

Ethical approval for this study was granted by the Ethics Committee of the Ruhr-University of Bochum (No. 21–7258) and supplemented by approvals from the local ethics committees of each participating center. Due to the retrospective design and de-identified nature of the data, the requirement for individual informed consent was waived. All methods were performed in accordance with the relevant guidelines and regulations.

### Patient outcomes

The analysis compared outcomes of patients admitted between March 2020 and May 2021 (period 1, encompassing the wild-type and the following virus variants: Alpha B.1.1.7 , Beta B.1.351) with those admitted from June 2021 to January 2022 (period 2, encompassing Delta B.1.617.2)^15,16^. The primary outcome was 30-day mortality following ICU admission. Secondary outcomes assessed included the ICU free days, days without ventilatory support (non-invasive, invasive, extracorporeal membrane oxygenation) or days without vasopressors, the rate of superinfections (bacterial, viral, and fungal), and the time from symptom onset to the development of critical disease and ICU admission. Furthermore, we compared the highest Murray Lung Injury Score^17^ and highest Sequential Organ Failure Assessment (SOFA) Score, over the course of the first 7 days of ICU stay. In addition, permanent medication was defined as the routine medication patients received independent of ICU treatment for COVID-19. Additionally, we documented the proportion of patients receiving adjuvant therapy, specifically screening for glucocorticoids, IL-6 receptor antagonists, remdesivir, and convalescent plasma therapy. An option to mark any additional treatments as “Other” was also included. Regarding immunization, vaccination status was categorized as no immunization, vaccinated, or recovered and reported as frequencies. The specific type of vaccine was not included in our common data set.

### Statistical analysis

Continuous variables are expressed as means ± standard deviation (SD) for normally distributed data and as medians with interquartile ranges (IQR; 25th to 75th percentile) for non-normally distributed data. Differences between groups for continuous variables were analyzed using the t-test or the Wilcoxon rank-sum test, based on the data distribution. Categorical variables were examined using the Chi-square test or Fisher’s exact test, as appropriate. Survival analysis was conducted using the Kaplan–Meier method to estimate time-dependent survival probabilities, with the log-rank test applied to evaluate differences between groups. Cox regression analysis was utilized to assess the impact of the pandemic period on survival outcomes, quantifying hazard ratios (HRs). Statistical significance was set at a p-value of less than 0.05, and confidence intervals (CIs) were set at 95%. All statistical analyses were performed using R software (version 3.5.3, R Foundation for Statistical Computing, Vienna, Austria). Figures were generated using the ggplot2 package in R.

## Results

### Baseline characteristics

A total of 447 critically ill COVID-19 patients were included, with 316 admitted during period 1 (March 2020–May 2021) and 131 in period 2 (June 2021–January 2022). Baseline characteristics, including age, Body mass index, and gender distribution, were comparable between the two periods (Table 1).

**Table 1:** Cohort description, classification according to period of pandemic.

	**Period 1** **(n = 316)**	**Period 2** **(n = 131)**	**p-value**
**Base characteristics**			
Female sex, n (%)	79(25%)	39 (30%)	0.346
Age, years (IQR)	61.0 (53–70)	59 (47.5–70)	0.110
BMI, kg/m^2^ (IQR)	30.0 (26.4–34.3)	29.5 (26.0–33.9)	0.535
SOFA score, day 1 (IQR)	7 (5–11)	6 (4–9)	**0.003**
Horowitz index, day 1 (IQR)	104.0(73.5–150.0)	97.5 (67.8–140.8)	0.143
Murray lung injury score, day 1 (IQR)	10(7–13)	8 (7–12)	**0.046**
Vasopressor support, day 1, n (%)	165(52%)	53(41%)	** < 0.001**
Acute kidney injury, day 1, n (%)			
No AKI	210(66%)	83(63%)	0.293
KDIGO 1	40(13%)	25(19%)	
KDIGO 2	20(6%)	11(8%)	
KDIGO 3	41(13%)	12(9%)	
**Comorbid conditions, n (%)**			
Hypertension	201 (63%)	62 (47%)	**0.002**
Cardiovascular disease	85 (27%)	25 (19%)	0.092
Chronic heart failure	31 (10%)	11 (8%)	0.724
COPD	23 (7%)	7 (5%)	0.538
Nicotine abuse	28 (9%)	11 (8%)	1.000
Solid organ transplantation	6 (2%)	2 (2%)	1.000
Chronic kidney disease	39 (12%)	17 (13%)	0.876
Diabetes mellitus	101 (32%)	41 (31%)	1.000
Malignant neoplasms	48 (15%)	26 (20%)	0.268
**Permanent medication, n (%)**			
ACE Inhibitors	77 (24%)	22 (17%)	0.103
AT1 Receptor blocker s	51 (16%)	18 (14%)	0.568
Beta blockers	106 (33%)	30 (23%)	**0.032**
Platelets inhibitors	76 (24%)	15 (11%)	**0.003**
Anticoagulants	31 (10%)	10 (8%)	0.590
Glucocorticoids	39 (12%)	7 (5%)	**0.027**
Immunosuppressants	25 (8%)	6 (5%)	0.305
Opioids	19 (6%)	3 (2%)	0.147
Total amount of medication	3 (0–7)	2 (0–6)	0.136
**Laboratory values, admission**			
Leucocytes [1000/µL]	11.3 (7.2–17.0)	10.2 (6.6–16.2)	0.150
C-reactive protein [mg/L]	25.73 (12.35–95.55)	28.6 (11.5–92.0)	0.833
Procalcitonin [ng/mL]	0.49 (0.20–1.32)	0.36 (0.15–1.02)	0.189
Interleukine-6 [pg/mL]	96.4 (40.2–286.1)	106.3 (45.3–493.6)	0.288
Ferritin [µg/L]	1241.5 (514.6–2096.1)	1283.0 (593.0–2397.0)	0.482
Lactate dehydrogenase [U/L]	462.0 (348.8–622.5)	519.5 (352.8–664.0)	0.355
Hemoglobin [g/dL]	10.6 (8.5–12.8)	10.8 (8.9–12.8)	0.431
Thrombocytes [1000/µL]	222.0 (158.5–295.0)	217.0 (152.0–290.0)	0.806
Bilirubin [mg/dL]	0.71 (0.40–2.00)	0.70 (0.40–2.50)	0.644
Creatinine [mg/dL]	1.16 (0.73–4.26)	1.13 (0.80–3.52)	0.545

*Data are presented as n (%) and median (IQR).*

In terms of the concomitant medication, beta blockers (33% vs. 23%, p = 0.032), platelet inhibitors (24% vs. 11%, p = 0.003), and glucocorticoids (12% vs. 5%, p = 0.027) were used more frequently in period 1. Other medications showed no significant differences. The total amount of medication was similar, with a median of 3 (IQR: 0–7) in period 1 and 2 (IQR: 0–6) in period 2 (p = 0.136).

Laboratory values at admission did not differ significantly between the periods for leukocytes, C-reactive protein, procalcitonin, interleukin-6, ferritin, lactate dehydrogenase, hemoglobin, thrombocytes, bilirubin, or creatinine (Table 1).

### Differences in outcomes with respect to the period of the pandemic

**Fig. 1:**
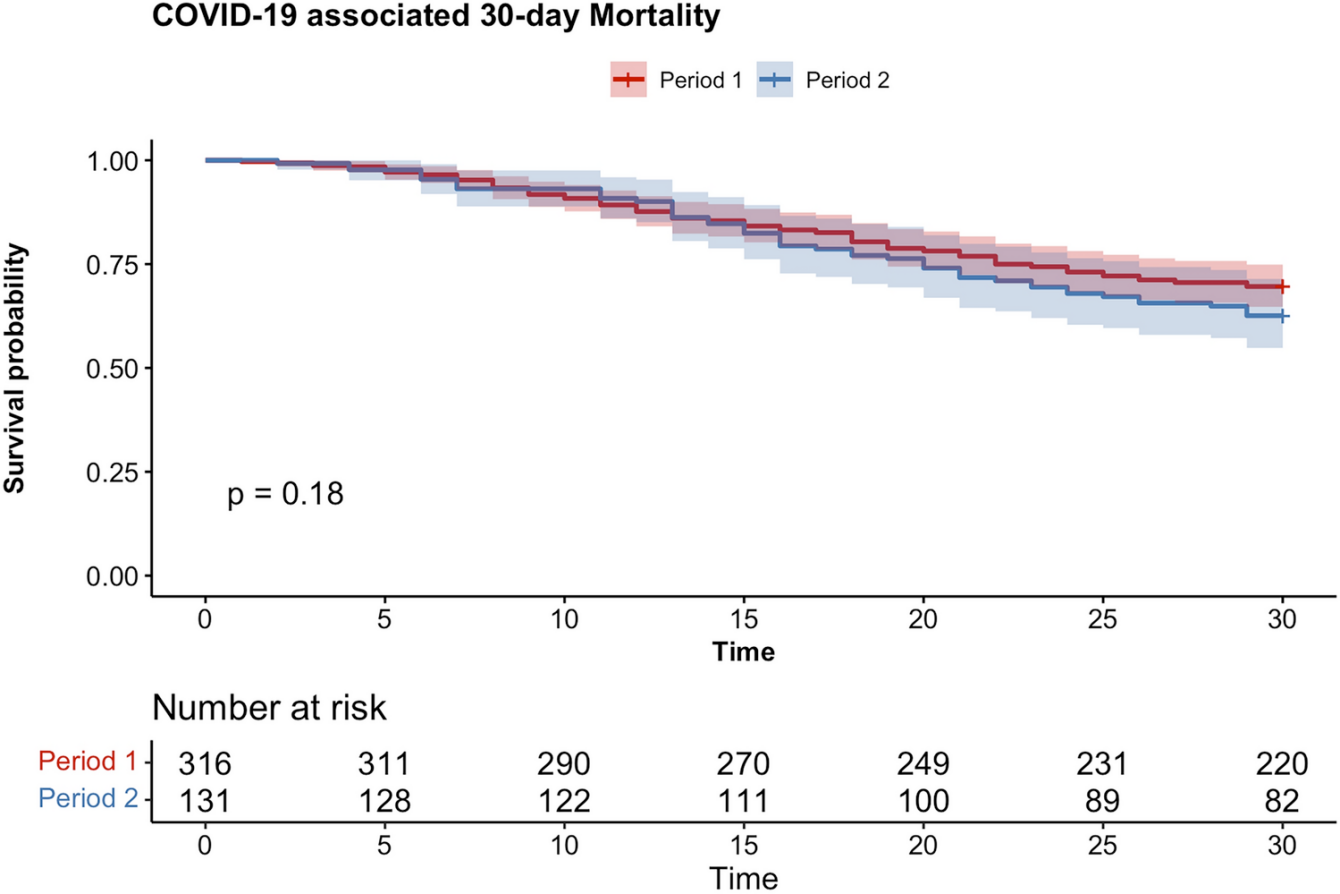
Kaplan–Meier Survival Analysis of critically ill COVID-19 patients (n = 447) admitted to an intensive care unit, stratified by period of the pandemic. The decrease in overall COVID-19-associated mortality over the course of the pandemic until January 2022 is not reflected in the mortality of severe COVID-19 cases. There was no significant difference with respect to the duration of the pandemic.

**Fig. 2:**
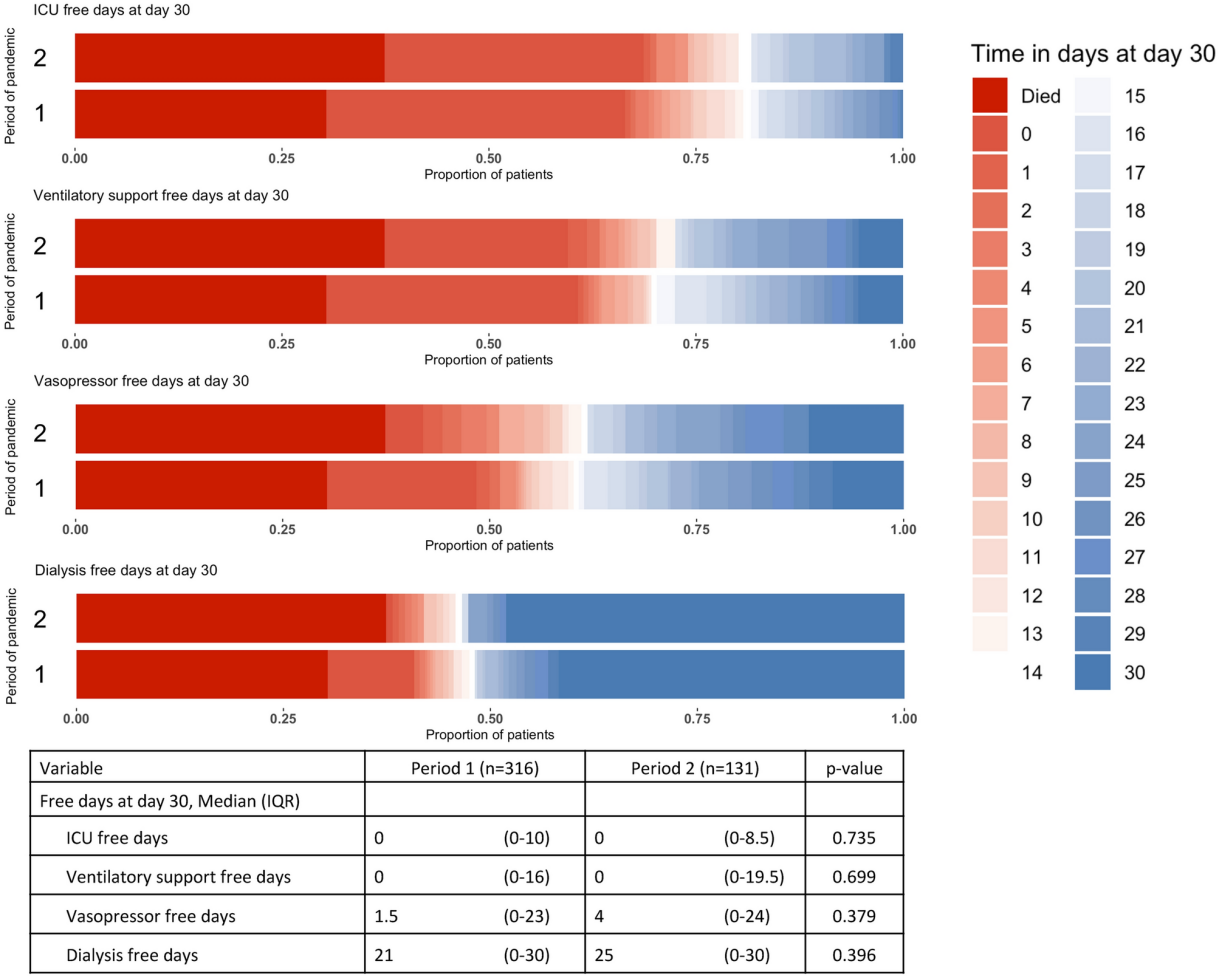
ICU, Ventilatory Support, Vasopressor and Dialysis Free Days at Day 30 During Different Periods of the Pandemic. This figure illustrates the distribution of ICU free days, ventilatory support free days, vasopressor free days and dialysis free days at day 30 for critically ill COVID-19 patients across both periods of the pandemic. Each panel shows the proportion of patients with a specific number of free days, color-coded from red (indicating death within 30 days) to blue (indicating 30 days). The table below summarizes the median and interquartile ranges (IQR) for these variables.

The outcomes of patients, classified according to the period of the pandemic, are summarized in Table 2. Despite advances in clinical experience and treatment strategies, 30-day mortality remained unchanged between the two periods with 30% (96 of 316 patients) in period 1 vs. 37% (39 of 131 patients) in period 2 (HR 1.26, 95% CI: 0.90–1.79, p = 0.181)(Fig. 1). Similarly, key ICU outcomes, including ICU-free days (p = 0.735), ventilatory support-free days (p = 0.699), vasopressor-free days (p = 0.379), and dialysis-free days (p = 0.396), did not show significant difference between the two periods (Fig. 2). The highest level of ventilatory support required showed also no significant difference between the periods for supplemental oxygen (2% vs. < 1%, p = 0.679), non-invasive ventilation (14% vs. 13%, p = 0.880), or mechanical ventilation (84% vs. 87%, p = 0.560).

**Table 2:** Patient outcome, classification according to period of pandemic.

	**Period 1** **(n = 316)**	**Period 2** **(n = 131)**	**p-value**
**Primary outcome**			
30-day mortality, n (%)	96(30%)	39 (37%)	0.181
**Secondary outcomes**			
ICU length of stay, days (IQR)	22 (12–38)	21 (12–35)	0.667
Ventilatory support, days (IQR)	17 (9–32)	15 (6–29)	**0.020**
Highest ventilatory sup port			
Supplemental oxygen, n (%)	6 (2%)	1 (< 1%)	0.679
Non-invasive ventilation, n (%)	44 (14%)	17 (13%)	0.880
Mechanical ventilation, n (%)	266 (84%)	113 (87%)	0.560
Highest lung injury score, (IQR)	13 (11–14)	12 (10–14)	**0.010**
Highest SOFA score, (IQR)	11 (8–14)	9 (7–12)	** < 0.001**
Superinfection, n (%)	248 (78%)	93 (71%)	0.112
Time between symptom onset and ICU admission, days (IQR)	9 (6–14)	7 (4–13)	**0.002**

*Data are presented as n (%) and median (IQR).*

Interestingly, although overall mortality from COVID-19 declined globally during later waves, our data suggest that once patients progressed to a critical stage, their prognosis did not significantly improve over time. This finding underscores the persistent vulnerability of a subset of ICU patients, despite increased availability of vaccines and evolving treatment strategies.

Although ICU mortality remained stable, disease severity at admission was higher in period 1 compared to period, as reflected by significantly higher SOFA scores (11, IQR: 8–14 vs 9, IQR: 7–12, p < 0.001) and Murray Lung Injury Scores (13, IQR: 11–14 vs 12, IQR: 10–14, p = 0.010). Patients in period 1 also had a longer time from symptom onset to ICU admission, potentially due to hospital admission constraints and delayed escalation of care in the earlier phase of the pandemic. Notably, these differences in disease severity did not translate into improved survival in period 2.

The rate of superinfection was not significantly different between the periods, with 78% (248 of 316 patients) in period 1 and 71% (93 of 131 patients) in period 2 (p = 0.112). The time between symptom onset and ICU admission was significantly shorter in period 2, with a median of 7 days (IQR: 4–13) compared to 9 days (IQR: 6–14) in period 1 (p = 0.002).

### Adjuvant therapy and immunization

Table 3 provides a summary of the adjuvant therapy and immunization status of patients during the two periods of the pandemic. The use of adjuvant glucocorticoid therapy was high in both periods, with 84% (264 of 316 patients) receiving this treatment in period 1 and 89% (116 of 131 patients) in period 2 (p = 0.193). The utilization of IL6-receptor antagonists slightly decreased from 11% (34 of 316 patients) in period 1 to 7% (9 of 131 patients) in period 2 (p = 0.223). Notably, the administration of remdesivir significantly declined from 18% (58 of 316 patients) in period 1 to 7% (9 of 131 patients) in period 2 (p < 0.001). Conversely, the use of convalescent plasma therapy increased from 7% (22 of 316 patients) in period 1 to 11% (15 of 131 patients) in period 2, although this change was not statistically significant (p = 0.132). By period 2, widespread vaccination had become available, yet 81% (106 of 131) patients remained unvaccinated, indicating that severe COVID-19 continued to disproportionately affect non-immunized individuals.

**Table 3:** Adjuvant therapy and immunization, classification according to period of pandemic.

	**Period 1** **(n = 316)**	**Period 2** **(n = 131)**	**p-value**
**Adjuvant therapy**			
Glucocorticoids	264 (84%)	116 (89%)	0.193
IL6-receptor antagonists	34 (11%)	9 (7%)	0.223
Remdesivir	58 (18%)	9 (7%)	** < 0.001**
Convalescent plasma therapy	22 (7%)	15 (11%)	0.132
other	109 (34%)	13 (10%)	** < 0.001**
**Immunization**			
No Immunization	316 (100%)	106 (81%)	** < 0.001**
Fully vaccinated	0 (0%)	25 (19%)	
Recovered	0 (0%)	0 (0%)	

*Data are presented as n (%) and median (IQR).*

Table 4 presents a comparative analysis of vaccinated (n = 25) and non-vaccinated (n = 106) patients during period 2 of the study. Vaccinated patients were significantly older, with a median age of 70 years (IQR: 63–76) compared to 57 years (IQR: 42.5–65.5) for non-vaccinated patients (p < 0.001). Additionally, vaccinated patients had a significantly higher prevalence of malignant neoplasms (44% vs. 14%, p = 0.002).

**Table 4:** Subgroup analysis of vaccinated versus non-vaccinated patients within period 2.

	**Vaccinated** **(n = 25)**	**Non-vaccinated** **(n = 106)**	**p-value**
**Selected base characteristics**			
Female sex, n (%)	4(16%)	35 (33%)	0.142
Age, years (IQR)	70 (63–76)	57 (42.5–65.5)	** < 0.001**
SOFA score, day 1 (IQR)	6 (4–7)	7 (4–9)	0.322
Horowitz index, day 1 (IQR)	105.0(76–160)	94.0 (67.5–139.0)	0.759
**Comorbid conditions, n (%)**			
Hypertension	12 (48%)	50 (47%)	1.000
Cardiovascular disease	6 (24%)	19 (18%)	0.572
Chronic heart failure	4 (16%)	7 (7%)	0.220
COPD	3 (12%)	4 (4%)	0.127
Nicotine abuse	4 (16%)	7 (7%)	1.000
Solid organ transplantation	1 (4%)	1 (< 1%)	0.347
Chronic kidney disease	6 (24%)	11 (10%)	0.094
Diabetes mellitus	8 (32%)	33 (31%)	1.000
Malignant neoplasms	11 (44%)	26 (14%)	**0.002**
**Primary outcome**			
30-day mortality, n (%)	9(36%)	40 (38%)	0.990
**Secondary outcomes**			
ICU length of stay, days (IQR)	13 (9–24)	21.5 (13–37)	**0.040**
Ventilatory support, days (IQR)	10 (4–22)	18.5 (7–30)	**0.021**
Highest ventilatory support			
Supplemental oxygen, n (%)	0 (0%)	1 (< 1%)	1.000
Non-invasive ventilation, n (%)	6 (24%)	11 (10%)	0.097
Mechanical ventilation, n (%)	19 (76%)	94 (90%)	0.097
Highest lung injury score, (IQR)	11 (8–13)	12 (10–14)	0.160
Highest SOFA score, (IQR)	9 (8–13)	9 (7–12)	0.429
Superinfection, n (%)	15 (60%)	78 (74%)	0.221
Time between symptom onset and ICU admission, days (IQR)	7 (4–9)	7 (4–13)	0.596
**Free days at day 30**			
ICU free days, (IQR)	0 (0–18)	0 (0–4)	0.091
Ventilatory support free days, (IQR)	1 (0–24	0 (0–13)	0.117
Vasopressor free days, (IQR)	7 (0–24)	3 (0–24)	0.351
Dialysis free days, (IQR)	30 (0–30)	24 (0–30)	0.667

*Data are presented as n (%) and median (IQR).*

Despite the aforementioned differences, the 30-day mortality rate was similar between vaccinated (36%) and non-vaccinated patients (38%) (p = 0.990). However, vaccinated patients experienced a shorter ICU length of stay (median 13 days, IQR: 9–24) compared to non-vaccinated patients (median 21.5 days, IQR: 13–37; p = 0.040). The duration of ventilatory support was also significantly shorter for vaccinated patients (median 10 days, IQR: 4–22) compared to non-vaccinated patients (median 18.5 days, IQR: 7–30) (p = 0.021). There were no significant differences in ICU free days, ventilatory support free days, vasopressor free days or dialysis free days at day 30 between the two groups. Taking these results into account, vaccination did not significantly impact 30-day mortality, although vaccinated patients required shorter ICU stays and less ventilatory support, suggesting potential benefits in disease progression rather than outright survival advantage.

## Discussion

While overall COVID-19 mortality has declined over the course of the pandemic, largely due to vaccination campaigns and improved treatment strategies, it remains unclear whether these advancements have translated into improved survival for critically ill COVID-19 patients on the ICU. Our study directly addresses this knowledge gap by specifically focusing on ICU patients, a population at persistently high risk of mortality despite evolving management approaches. By analyzing ICU admissions across two distinct pandemic periods, we assessed whether 30-day mortality in critically ill COVID-19 patients decreased over time, independent of changes in disease severity or treatment protocols.

Notably, despite improved clinical experience and the widespread availability of vaccines in period 2 (from June 2021 to January 2022), mortality rates remained unchanged between the two periods, suggesting that critical illness from COVID-19 continues to carry a substantial fatality risk, irrespective of broader public health improvements. This finding contrasts with epidemiological studies that have shown a significant reduction in overall COVID-19-associated mortality over time^9^. These studies attributed this trend mainly to factors such as the global vaccination campaign and novel treatment options, which mainly contributed to preventing the occurrence of a severe to critical course of COVID-19^18–20^. However, our data compellingly show that these factors did not impact mortality once COVID-19 progressed into a critical course. Therefore, we must acknowledge that vulnerable subgroups of patients remain prone to fatal complications caused by SARS-CoV-2 infections. As this finding may initially seem controversial, our focus was on a detailed description of the two cohorts to investigate whether there were any specific factors contributing to this observation.

A priori, we expected and hypothesized that patients in the later phase (period 2) of the pandemic would exhibit lower mortality rates also for critical COVID-19 itself due to increased clinical experience and clearer evidence-based treatment approaches for managing these patients^21,22^. This expectation is strengthened by the fact that patients in our study demonstrated higher disease severity both at the time of admission and throughout their ICU stay during the early phase (period 1), as evidenced by significantly higher SOFA scores and Murray Lung Injury Scores at admission and their peak levels. Additionally, a greater proportion of patients in the early phase required vasopressors upon ICU admission compared to those in the later period. And finally, time between the onset of symptoms and ICU admission also differed significantly, being shorter in the later phase of the pandemic. Here, two factors might be responsible for this observation. First, it is known that the dynamics of the disease changed, with a shorter incubation period as the pandemic progressed^23,24^. Additionally, this may reflect a successful strategy for early detection and increased vigilance regarding COVID-19 symptoms^25^. On the other hand, it is important to consider that during the early phase of the pandemic, there was significant concern about shortages in ICU capacity^4,5^. Consequently, hospital and ICU admissions were often delayed, which aligns with the observation that patients in the early phase of the pandemic exhibited greater disease severity upon ICU admission, with higher SOFA scores and more frequent need for vasopressors. Nevertheless, this did not translate into an association with 30-day survival.

So, at first glance, our results appear contradictory. One would expect a reduction in disease severity and mortality, particularly given the widespread availability of the vaccine, but our findings did not support this expectation. However, our study’s observation of consistent 30-day ICU mortality rates across different periods aligns with the French study, which also reported stable ICU mortality despite changes in treatment protocols^26^.

Regarding immunization, a nuanced interpretation is required. While vaccination was not available during the early phase and population immunity was low, the situation changed in December 2020 with the introduction of vaccines^27^. Interestingly, 81% of patients in the later period of our study (June 2021 to January 2022) remained unvaccinated. Meanwhile, by mid of 2021, less than 40% of the general population in Germany and Austria were still not vaccinated^28^. Therefore, our cohort shows a dramatic overrepresentation of unvaccinated patients among critically ill COVID-19 cases. When examining the subgroup of non-immunized, our study found that unvaccinated patients in period 2 were significantly younger than vaccinated patients, raising the question of whether this shift reflects the protective impact of vaccination in preventing severe disease among higher-risk populations or is a result of targeted immunization policies^29^. The demographic shift towards younger, unvaccinated patients in later periods mirrors findings from other studies, indicating that vaccination efforts initially prioritized older and high-risk populations, thereby reducing their representation among critically ill patients in subsequent waves^30,31^. Thus, the overrepresentation of younger, unvaccinated patients in period 2 aligns with broader epidemiological trends showing that COVID-19 ICU admissions became increasingly concentrated in non-immunized individuals as vaccination programs progressed. By mid-2021, a substantial proportion of the general population in Germany and Austria had received at least one vaccine dose. However, our study cohort demonstrates that the vast majority of critically ill COVID-19 patients in ICUs remained unvaccinated, emphasizing that vaccine hesitancy and lack of immunization were major risk factors for severe disease requiring ICU admission.

Early vaccination campaigns in Germany and Austria also prioritized high-risk groups, including older adults and individuals with pre-existing conditions, particularly those with malignancies and immunosuppressive disorders. This prioritization likely contributed to the observed age difference, as these individuals were more likely to be vaccinated and still at risk of severe COVID-19 despite immunization. Of note, vaccinated patients who nevertheless developed a critical course of COVID-19 showed a dramatically higher prevalence of oncological comorbidities. Thus, patients suffering from malignancies, particularly haemato-oncological patients, represent a subgroup that seems particularly at risk despite vaccination. The crucial question of whether this is due to reduced vaccine efficacy from inherently low immunocompetence or evoked by other co-factors associated with the malignant disease is beyond the scope of this study. Therefore, the immunological profiles of patients with a higher risk of experiencing critical COVID-19 should be explored in future research to address this important question. Additionally, in the context of future pandemics and public health interventions, this group requires special protection despite the availability of effective vaccines.

Summarizing, our findings suggest that two distinct phenomena shaped ICU admissions in the later phase of the pandemic: (1) a protective shift, where vaccination reduced the likelihood of severe disease in lower-risk populations, leading to fewer ICU admissions in vaccinated individuals, and (2) a selective effect, where vaccinated patients who nevertheless developed critical illness were predominantly those with underlying malignancies or immunosuppression, reflecting their persistent vulnerability despite immunization.

In summary, our data indicate that once COVID-19 progresses to a critical stage, advances in treatment protocols and availability of novel therapeutics have not translated into improved survival rates in critical COVID-19. The persistently high mortality rate in unvaccinated patients, as they are highly comparable to unvaccinated patients in period one, suggests that once crossed an immunological or pathophysiological threshold, elaborate intensive care treatments are still less effective. This is similar to bacterial sepsis and addressing this challenge may require a phenotyping approach or systemic immunological characterization^32,33^. Such detailed analysis could uncover critical insights into the mechanisms driving severe outcomes and identify novel targets for personalized therapeutic interventions.

### Limitations

This study has some limitations. The generalizability of our findings is constrained by the fact that the study was conducted across a limited number of centers in Germany and Austria, which may not be representative for lower income countries and regions. Additionally, despite efforts to standardize treatment across study centers, variations in the management of critical COVID-19 cases, such as the timing and application of mechanical ventilation and administration of medications, were not fully standardized between the different centers. As this is a retrospective study, the accuracy of our data relies heavily on the quality of documentation in medical records, and potential inaccuracies or missing data could affect the validity of our findings. Furthermore, there may be unmeasured confounding factors, such as socioeconomic status, pre-existing health conditions not captured in the study, and variations in supportive care, that could influence the outcomes observed. In addition, one key limitation is the sample size, particularly the smaller number of patients in period 2 (131 vs. 316 in period 1), which may have reduced statistical power and the ability to detect subtle mortality trends. This imbalance reflects the natural decline in ICU admissions as vaccination rates increased and case severity decreased. Additionally, the modest cohort size limits the generalizability of our findings to broader ICU populations. Nonetheless, our study provides valuable real-world data on persistent ICU mortality in critically ill COVID-19 patients. Finally, we stopped data collection with the emergence of the Omicron variant^15,34^. Therefore, assessing the impact of all virus variants following Delta is beyond the scope of this study.

## Conclusion

Patients admitted to the ICU for critical COVID-19 at the beginning of the pandemic exhibited higher disease severity. Nevertheless, and despite advancements in COVID-19 recognition, treatment, and immunization, our study found that mortality among critically ill COVID-19 patients in ICUs did not significantly decrease over the course of the pandemic. Our data indicate that a highly vulnerable group of patients remains at dramatically increased risk of severe complications. Further research is essential to better understand the underlying factors contributing to this persistent susceptibility.

## Data Availability

The dataset analyzed during the current study is available from the corresponding author on reasonable request.

## Abbreviations

ACE: Angiotensin-Converting Enzyme
ARDS: Acute Respiratory Distress Syndrome
AT1: Angiotensin II Type 1
CI: Confidence Interval
COPD: Chronic Obstructive Pulmonary Disease
COVID-19: Coronavirus Disease 2019
FiO2: Fractional Inspired Oxygen
HR: Hazard Ratio
ICU: Intensive Care Unit
IQR: Interquartile Range
KDIGO: Kidney Disease Improving Global Outcomes
mRNA: Messenger Ribonucleic Acid
paO2: Arterial Oxygen Partial Pressure
SD: Standard Deviation
SOFA: Sequential Organ Failure Assessment
WHO: World Health Organization
